# Protocol for the Ketamine for Postoperative Avoidance of Depressive Symptoms (K-PASS) feasibility study: A randomized clinical trial

**DOI:** 10.12688/f1000research.121529.1

**Published:** 2022-05-11

**Authors:** Bradley A. Fritz, Bethany R. Tellor Pennington, Ben J.A. Palanca, Julie A. Schweiger, Jon T. Willie, Nuri B. Farber

**Affiliations:** 1 Department of Anesthesiology, Washington University in St. Louis, St. Louis, Missouri, 63110, USA; 2 Department of Psychiatry, Washington University in St. Louis, St. Louis, Missouri, 63110, USA; 3 Department of Neurosurgery, Washington University in St. Louis, St. Louis, Missouri, 63110, USA

**Keywords:** Depression, Feasibility, Ketamine, Protocol, Randomized Clinical Trial, Surgery

## Abstract

**Background: **Postoperative depressive symptoms are associated with pain, readmissions, death, and other undesirable outcomes. Ketamine produces rapid but transient antidepressant effects in the perioperative setting. Longer infusions confer lasting antidepressant activity in patients with treatment-resistant depression, but it is unknown whether a similar approach may produce a lasting antidepressant effect after surgery. This protocol describes a pilot study that will assess the feasibility of conducting a larger scale randomized clinical trial addressing this knowledge gap.

**Methods: **This single-center, double-blind, placebo-controlled pilot trial involves the enrollment of 32 patients aged 18 years or older with a history of depression scheduled for surgery with planned intensive care unit admission. On the first day following surgery and extubation, participants will be randomized to an intravenous eight-hour infusion of either ketamine (0.5 mg kg ^-1^ over 10 minutes followed by a continuous rate of 0.3 mg kg ^-1^ h ^-1^) or an equal volume of normal saline. Depressive symptoms will be quantified using the Montgomery-Asberg Depression Rating Scale preoperatively and serially up to 14 days after the infusion. To detect ketamine-induced changes on overnight sleep architecture, a wireless headband will be used to record electroencephalograms preoperatively, during the study infusion, and after infusion. The primary feasibility endpoints will include the fraction of patients approached who enroll, the fraction of randomized patients who complete the study infusion, and the fraction of randomized patients who complete outcome data collection.

**Conclusions: **This pilot study will evaluate the feasibility of a future large comparative effectiveness trial of ketamine to reduce depressive symptoms in postsurgical patients.

**Registration:** K-PASS is registered on ClinicalTrials.gov: NCT05233566; registered February 10, 2022.

## Introduction

### Postoperative depression

Approximately 25-50% of patients presenting for surgery have a history of depression. ^1^^–^^5^ Patients with a history of preoperative depression are at elevated risk for experiencing depressive symptoms after surgery. In a cohort of 248 neurosurgical patients, lifetime history of depression was an independent predictor of postoperative depressive symptoms. ^6^ Similar risks have been observed in cardiac surgery. ^1^ When scores on various depression scales are analyzed as continuous variables, worse preoperative scores have consistently been significant predictors of worse postoperative scores. ^7^^–^^9^ Postoperative depressive symptoms of greater severity have been linked to increased pain, ^10^ more frequent hospital readmissions within six months, ^11^ as well as increased mortality in short-term and long-term follow-up. ^12^

Currently, prevention and treatment of depressive symptoms generally focuses on continuation, resumption, or initiation of oral antidepressants. The first-line agents include selective serotonin reuptake inhibitors and serotonin norepinephrine reuptake inhibitors, based on American Psychiatric Association recommendations for treatment of depression in adults and older adults. ^13^ Following initiation or dose titration, these medications take several weeks to achieve their full effect, ^14^^–^^16^ limiting their effectiveness to prevent acute depressive symptoms after surgery. Furthermore, it may not be possible to give these medications in some instances due to impaired gastrointestinal absorption or motility or due to concern for medication interactions such as serotonin syndrome. Given that rehabilitation is a common need following major surgery, rapid-acting antidepressants may be both more impactful and more easily administered in the perioperative compared to outpatient setting.

### Ketamine as an antidepressant

The N-methyl-D-aspartate (NMDA) receptor antagonist ketamine has shown promise as a novel, rapid-acting therapy for treatment-resistant depression. In 2006, Zarate and colleagues published an 18-patient randomized trial that demonstrated improved Hamilton Depression Rating Scale scores following 0.5 mg kg ^-1^ ketamine infused over 40 minutes compared to placebo. ^17^ Since then, this finding has been replicated multiple times, with a recent meta-analysis of 19 studies reporting that a single infusion of ketamine led to improved depressive symptoms compared to placebo at four hours and 24 hours. ^18^ Additional trials have demonstrated superiority of ketamine compared to active comparators such as midazolam for treatment of treatment-resistant depression. ^19^^,^^20^

The antidepressant properties of ketamine are potentially mediated by a sequence of events that lead to synaptogenesis and increased electroencephalogram (EEG) sleep slow wave activity (SWA). Ketamine administration results in increased extracellular glutamate in the prefrontal cortex, ^21^^–^^23^ initiating a chain of events ^24^^,^^25^ that leads to increased prefrontal synaptic density. ^26^ Enhanced synapse creation during wakefulness is followed by enhanced synapse pruning during subsequent sleep, for which sleep SWA (1-4 Hz total EEG power) during non-rapid eye movement (NREM) sleep is a commonly used surrogate. ^27^ Additionally, low sleep SWA during baseline sleep predicts ketamine responsiveness in depressed patients, ^28^ and increases in sleep SWA following ketamine infusion correlate with symptom response. ^29^

Although ketamine achieves its antidepressant effect rapidly, the effect of a single bolus wanes over the first week, and strategies to achieve longer effects have been explored, such as repeat intravenous boluses and intranasal administration. ^30^^–^^34^ Symptoms can be controlled for multiple weeks, but ongoing therapy is needed to sustain the effect. ^35^ An alternative strategy that reduces repeated dosing is to use a long-duration of infusion. In a pilot study, a 96-hour ketamine infusion titrated to a goal of 0.6 mg kg ^-1^ h ^-1^ led to a rapid reduction in depressive symptoms in a large majority of responders sustained for up to eight weeks. ^36^^,^^37^

Lessons learned from treatment-resistant depression may guide the mitigation of depressive symptoms in the perioperative arena. Ketamine is already familiar in this setting because it is sometimes used to provide sedation or to augment analgesia. ^38^^,^^39^ Boluses of 0.25-0.5 mg kg ^-1^ during Cesarean section have been associated with either no effect ^40^ or with reductions in postpartum depression. ^41^^,^^42^ In surgery with general anesthesia, ketamine boluses near the time of induction have been associated with reduced postoperative depressive symptoms among patients with a history of depression ^43^^,^^44^ but not in a general population of older adults. ^45^ In those studies where a significant beneficial effect was observed, the effect quickly waned over the first few days after surgery. Treatment strategies that produce longer-lasting effects are desirable, but the longer-duration infusion that has shown promise in treatment-resistant depression has not been tested in the postoperative setting.

### Hypothesis and objectives

The aim of this pilot study is to assess the feasibility of conducting a phase three clinical trial, which will test the hypothesis that a postoperative sustained, low-dose ketamine infusion can prevent postoperative depressive symptoms in surgical patients with a history of depression. Therefore, this feasibility trial has three primary objectives: to evaluate the feasibility of recruiting participants to the randomized trial, to evaluate the feasibility of delivering the study medication, and to evaluate the feasibility of collecting depression outcome data. Secondary objectives are to estimate the effect sizes for two efficacy outcomes: postoperative depressive symptoms as quantified using the Montgomery-Asberg Depression Rating Scale on post-infusion day four and delta sleep ratio on EEG collected the first night following the study medication.

## Methods

### Overall trial design

**Figure 1.**
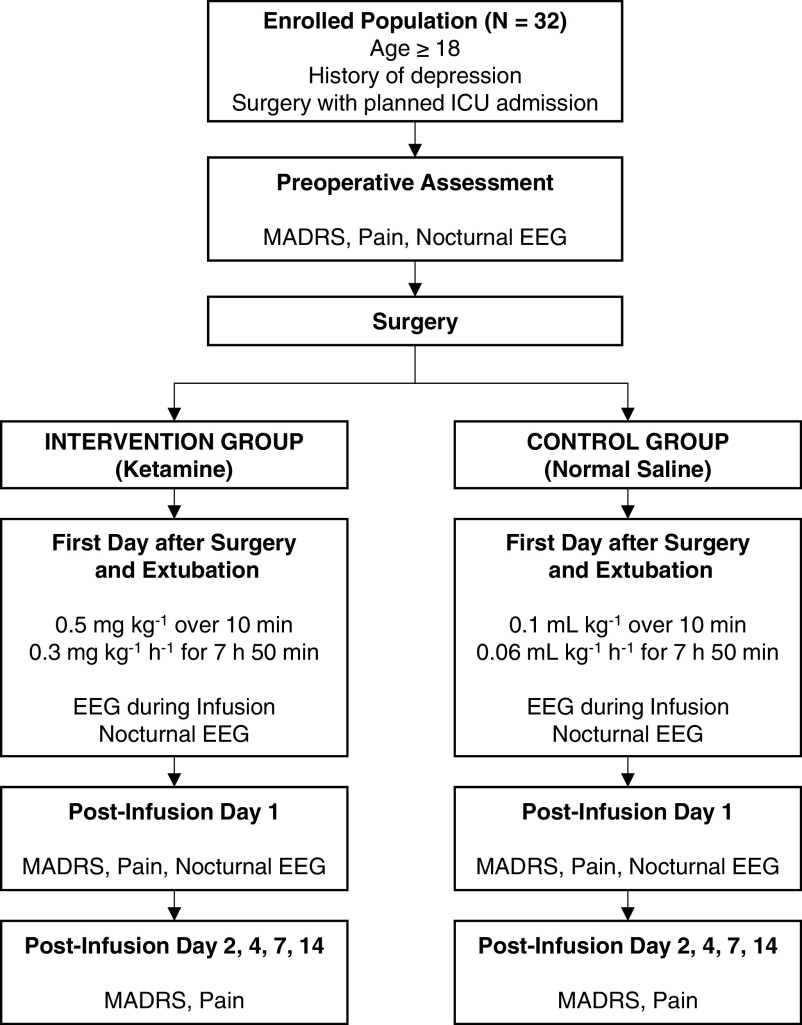
Flow chart of patient activities in the trial. Abbreviations: ICU = intensive care unit; MADRS = Montgomery-Asberg Depression Rating Scale; EEG = electroencephalogram.

This trial will follow a randomized, placebo-controlled, double-blinded, parallel design. It will be conducted at Washington University in St. Louis School of Medicine/Barnes-Jewish Hospital, a large academic hospital that serves as a quaternary referral center for a multi-state area in the Midwest region of the United States. The overall trial structure is shown in Figure 1. This protocol has been designed in accordance with the Standard Protocol Items: Recommendations for Interventional Trials (SPIRIT) guidelines. ^46^

### Eligibility criteria

Inclusion criteria for this trial will include patients (1) aged 18 years or older, (2) scheduled for surgery with planned intensive care unit (ICU) admission at Barnes-Jewish Hospital, (3) past medical history of depression, (4) ability to provide written, informed consent, and (5) stated willingness to comply with all study procedures. Past medical history of depression will be defined by previous diagnosis documented in the electronic health record or by previous use of an oral antidepressant.

Exclusion criteria will include (1) bipolar depression, (2) outpatient antipsychotic medication use, (3) emergent surgery, (4) known or suspected elevation in intracranial pressure, (5) subarachnoid hemorrhage, (6) carotid endarterectomy or arteriovenous malformation repair, (7) allergy to ketamine, (8) any condition in which a significant elevation of blood pressure would constitute a serious hazard (e.g., aortic dissection, pheochromocytoma), (9) outpatient use of an anticonvulsant with significant voltage-gated sodium channel activity, ^47^ (10) known history of dementia, (11) pregnancy or lactation, (12) inability to converse in English, (13) concurrent enrollment in another interventional trial, and (14) postoperative mechanical ventilation continuing past 07:00am on postoperative day three.

***Recruitment strategy***

Patients will primarily be recruited at the Center for Preoperative Assessment and Planning (CPAP). Potentially eligible patents will be identified by screening the CPAP schedule. Patients identified via screening will be approached by telephone prior to their CPAP appointment or in person at CPAP. In addition, the operating room schedule will be reviewed to identify potentially eligible patients admitted to the hospital before surgery who did not visit CPAP. These patients will be approached in person in the hospital prior to the day of surgery.

### Intervention

On the first morning following surgery and extubation, participants will be randomized in a 1:1 fashion to receive either ketamine or normal saline, in blocks of four. The randomization scheme will be held by the Barnes-Jewish Hospital Investigational Drug Service. Participants, research team members, and clinical staff (ICU nurses, physicians, pharmacists) will be blinded to treatment allocation. To facilitate blinding, study medication for both groups will be delivered in 100 mL bags with identical appearance except for a unique study identifier. The adequacy of blinding will be assessed on post-infusion day one by asking the participant to guess which treatment they received. The attending intensivist may request unblinding if a serious adverse event occurs and the intensivist feels knowledge of the treatment allocation will impact clinical decision making.

The study medication will be administered as a bolus loading dose followed by a continuous infusion. For participants in the ketamine group, the loading dose will consist of ketamine 0.5 mg kg ^-1^ administered intravenously over 10 minutes (or over 20 minutes if body mass index > 40). This will be followed by a continuous infusion at 0.3 mg kg ^-1^ h ^-1^ for an additional 7 hours 50 minutes. In the control group, an equal volume of normal saline will be administered over the same infusion rate schedule as for the ketamine group (loading dose of 0.1 mL kg ^-1^ followed by a continuous infusion at 0.06 mL kg ^-1^ h ^-1^). Study intervention dosing will be based on actual body weight. The study medication will be initiated between 05:00am and 08:00am. To improve adherence to intervention protocols, detailed instructions will be available in the electronic health record and on a laminated card given to the ICU nurse. In addition, research staff will check on the participant every few hours and be available throughout the infusion. Adherence will be monitored by reviewing the medication administration record in the electronic health record.

***Justification for dose***

In the intervention group, the dosing regimen has been designed to quickly achieve an expected blood level of ketamine of 225 ng mL ^-1^, with maintenance of that level throughout the infusion. A targeted blood level of 225 ng mL ^-1^ represents half of the serum concentration obtained during steady state for a 96-hour infusion. ^36^ This level is also only slightly higher than the peak blood level obtained after patients receive a 0.5 mg kg ^-1^ bolus dose delivered over 40 minutes, first described by Krystal in 1994 ^48^ and used frequently in subsequent studies. Such a dosing approach would address the question of clinical utility in prolonging the length of anticipated NMDA receptor blockade from several minutes to several hours.

To load the patient with an amount equal to 100% of the desired steady state, the loading dose should be equal to the product of the volume of distribution and the desired plasma concentration. Per Wagner and O’Hara, ^49^ the volume of distribution of ketamine is 2.4 L kg ^-1^. As noted above, the desired plasma concentration is 225 ng mL ^-1^, which is equal to 0.225 mg L ^-1^. Thus, the loading dose is 2.4 L kg ^-1^ × 0.225 mg L ^-1^ = approximately 0.5 mg kg ^-1^.

The dose for the continuous infusion is based on a previous study of 23 patients with treatment-resistant depression who received a 96-hour infusion of intravenous ketamine; serum ketamine levels were directly measured. ^37^ An infusion of 0.6 mg kg ^-1^ h ^-1^ produced a steady-state blood level of about 450 ng mL ^-1^. Therefore, an infusion at half that rate (0.3 mg kg ^-1^ h ^-1^) would be expected to produce a 50% lower steady-state blood level of 225 ng mL ^-1^.

***Dose adjustments***

Richmond Agitation and Sedation Scale (RASS) scores will be monitored per standard ICU nursing protocols. ^50^ If the participant experiences sedation to a RASS score ≤ -2, then the study medication will be halted until recovery to RASS > -2. Then the infusion will be resumed at a reduced rate. If the RASS score is ≤ -2 before initiation of the study medication, then the study medication will not be initiated at that time. The participant will be re-evaluated the following day, and the study medication will be initiated when the RASS is no longer ≤ -2. If RASS remains ≤ -2 on postoperative day three, the patient will be withdrawn from the study.

***Concurrent therapy***

Participants should not receive open-label ketamine at any time intraoperatively or postoperatively. Otherwise, all components of the anesthetic care plan will be at the discretion of the attending anesthesiologist. Postoperatively during the eight-hour study infusion, concurrent infusions of propofol, midazolam, or other sedative agents with gamma-aminobutyric acid (GABA) receptor activity will not be permitted. Concurrent dexmedetomidine will be permitted, but administration of benzodiazepines or gabapentin will not be permitted. All other components of postoperative care will occur as directed by the clinical team.

### Data collection

All data points will be collected for all patients, including those who deviate from the intervention protocols. To promote participant retention and complete follow-up, postoperative assessments can be made via telephone if the patient discharges from the hospital before the day of the final study assessment.

***Baseline factors potentially related to depression***

At the time of enrollment, the patient will complete several surveys evaluating factors that may be related to their depressive history or that may predict treatment responsiveness. These surveys will include the Generalized Anxiety Disorder 7-item scale (GAD-7), ^51^ the Alcohol Use Disorders Identification Test (AUDIT), ^52^ the Drug Abuse Screening Test (DAST-10), ^53^ and the Childhood Trauma Questionnaire (CTQ). ^54^

***Depressive symptoms***

Depressive symptoms will be measured using the Montgomery-Asberg Depression Rating Scale (MADRS) preoperatively and on post-infusion days 1, 2, 4, 7, and 14. The MADRS rates the severity of 10 depressive symptoms based on a targeted clinical interview, yielding a total score zero (no symptoms) and 60 (severe symptoms). ^55^^,^^56^ The scale has been found to correlate highly with a global clinician assessment (Pearson correlation = 0.71) ^57^ and to have high inter-rater reliability. ^55^ The MADRS has been modified to assess symptoms over the previous day rather than the previous week. ^36^^,^^37^ This modified version will be used on the post-infusion days.

The MADRS will be administered by a trained research team member. Post-infusion assessments will be conducted between 07:00am and 12:00pm on the specified days. The time of day for the preoperative assessment will be variable, depending on the time of the patient’s CPAP appointment or in-hospital visit. If the patient is discharged from the hospital before the day of the final study assessment, the MADRS will be conducted over the telephone.

Concurrent with each MADRS assessment, the research team member will use the Clinical Global Impression-Severity scale (CGI-S) to rate the overall severity of the patient’s mental illness. ^58^ At each postoperative follow-up, the Clinical Global Impression-Improvement scale (CGI-I) will also be used to rate the overall level of improvement compared to the previous assessment. Concurrent with each MADRS assessment, self-reported depressive symptoms will be collected using the self-report version of the Quick Inventory of Depressive Symptomatology (QIDS-SR). ^59^

The MADRS includes questions about suicidal ideation. If the patient reports any suicidal ideation, then the Suicide Risk Management Protocol will be initiated. This includes a structured assessment allowing stratification of the risk for self-harm, notification of the principal investigator, and (if risk is moderate or high) notification of an on-call psychiatrist.

***EEG measurement***

EEG will be captured using the Dreem headband (DREEM, Rhythm, New York, NY), a consumer-grade wireless device using dry electrodes. The device uses five EEG channels (F7, F8, FpZ, O1, O2), accelerometry, and pulse plethysmography. It samples EEG electrode potentials at a rate of 250 Hz and applies a 0.4-30 Hz bandpass filter.

At the baseline visit, the research team member will teach the participant how to use the Dreem headband for data collection. If the participant is recruited in the CPAP clinic, then the participant will take the Dreem headband home with them. They will be instructed to wear the headband and activate data collection while sleeping for one night. They will bring the headband back to the hospital with them on the day of surgery. If the participant is recruited in the hospital, then the participant will wear the headband and activate data collection while sleeping the night following enrollment. The headband will be retrieved the following day.

Additional data collection using the Dreem headband will occur during the study medication infusion, the night following the study medication infusion, and the night following post-infusion day one. Each EEG will be analyzed by an experienced sleep technician to identify sleep stages. Within each time epoch captured, SWA will be defined as the total power in the 1-4 Hz frequency band. The delta sleep ratio will be defined as the ratio of SWA during the first NREM Stage three cycle to SWA during the second NREM Stage three cycle.

***Postoperative pain***

Pain will be measured at the baseline visit and on post-infusion days 1, 2, 4, 7, and 14 using the visual analog scale and an 11-point numeric rating scale. The participant will rate their pain at rest, when taking a deep breath, and with movement. If the participant is discharged from the hospital before the day of the final study assessment, remaining pain assessments will be conducted over the telephone using the numeric rating scale only.

***Postoperative delirium***

Delirium is assessed routinely by the ICU nurses using the Confusion Assessment Method for the ICU (CAM-ICU) once per shift. ^60^ CAM-ICU scores between the day of the infusion and post-infusion day five will be retrieved from the electronic health record.

### Safety outcomes

***Psychotomimetic side effects***

Psychotomimetic effects will be quantified using the Brief Psychiatric Rating Scale (BPRS) four-item positive symptom subscale ^36^^,^^61^ and using the modified seven-item Clinical Administered Dissociative State Scale (CADSS-7). ^62^ The BPRS four-item subscale yields a score between four (no symptoms) and 28 (extremely severe). The CADSS-7 yields a score between zero (no symptoms) and 35 (severe symptoms). Both scales will be administered by a research team member midway through the study medication infusion (approximately four hours +/- one hour after the start of the bolus loading dose).

***Clinical and adverse events checklist***

Additional side effects of ketamine will be monitored using the Clinical and Adverse Events Checklist. ^63^ This checklist will be administered by a research team member midway through the study medication infusion (approximately four hours +/- one hour after the start of the bolus loading dose). The checklist will be repeated on post-infusion day 14 to monitor for resolution of any side effects.

***Vital signs during study medication infusion***

During the infusion, participants will receive vital sign monitoring per ICU standard of care. This will include continuous telemetry, continuous pulse oximetry, and either intermittent noninvasive blood pressure (at least hourly) or continuous invasive blood pressure (if an arterial catheter is present). Vital signs will be documented in the electronic health record by the clinical bedside nurse per unit standard of care. The research team will retrieve vital signs from the electronic health record.

Safety events will include significant hypertension during the infusion, defined as systolic blood pressure > 180 mmHg or the administration of an antihypertensive medication that is not one of the patient’s home medications. Another safety event will be tachycardia, defined as heart rate > 100 beats per minute, during the infusion.

***Nausea and vomiting***

At each post-infusion study visit, participants will be asked if they have experienced nausea or vomiting in the past 24 hours. If present, the patient will be asked to classify the event as mild, moderate, or severe. In addition, the electronic medical record will be reviewed for administration of antiemetic medication.

### Statistical methods

***Primary endpoints***

For primary objective one, the endpoint will be the fraction of approached patients who enroll in the trial and are randomized. This endpoint will be described using a proportion and 95% confidence interval. The numerator will include all participants who are randomized to receive either ketamine or the placebo. The denominator will include all patients who are approached by the research team in person or by telephone to evaluate eligibility and offer consent.

For primary objective two, the endpoint will be the fraction of randomized participants who complete the study medication infusion. This endpoint will be described using a proportion and 95% confidence interval. The numerator will include all participants who receive the study medication for at least seven of the planned eight hours. The denominator will include all participants who are randomized.

For primary objective three, the endpoint will be the fraction of randomized participants who have MADRS scores available at all the specified time points. This endpoint will be described using a proportion and 95% confidence interval. The numerator will include all participants with MADRS scores documented ate baseline and post-infusions days 1, 2, 4, 7, and 14. The denominator will include all participants who are randomized.

***Secondary endpoints***

For secondary objective one, the endpoint will be the difference in MADRS score on post-infusion day four compared to the preoperative baseline visit. Participants with missing MADRS scores at either time point will be excluded. For secondary objective two, the endpoint will be the EEG delta sleep ratio on the second night following the study medication infusion. Point estimates and standard deviations for the between-group differences in these endpoints will be obtained, but this pilot study will not be powered to perform formal statistical testing. These analyses will follow the intention-to-treat principle. There will be no imputation of missing data.

***Sample size***

The sample size of 32 has been selected to allow the primary descriptive endpoints to be measured with acceptable levels of precision. For endpoint one, if 25% of approached patients enroll (which is a conservative estimate), then this sample size will allow this proportion to be measured with a 95% confidence interval width of ±15%. For endpoint two, if 90% of randomized participants complete the study medication infusion, then this sample size will allow this proportion to be measured with a 95% confidence interval width of ±10%. For endpoint three, if 95% of randomized participants have MADRS scores available at all specified time points, then this sample size will allow this proportion to be measured with a 95% confidence interval of ±7%.

This sample size does not provide power to test the secondary endpoints for superiority. However, the observed values will be used to determine effect sizes, as well as standard deviations for the observed values. These quantities will inform the sample size calculation for the full-scale clinical trial. A minimum of 24-30 patients has previously been recommended for estimating effects sizes, ^64^^,^^65^ so this feasibility trial should be large enough to provide the necessary estimates.

### Safety monitoring

This trial will employ an independent safety monitor (ISM) to serve as an impartial advocate for the safety of study participants. The ISM will be a physician with relevant expertise in the care of postoperative critically ill patients and must be independent from the study conduct. Any reportable adverse events (including unexpected adverse drug events and unanticipated problems) will be reported to the ISM and to the institutional review board within one business day if it involves a death or within 10 business days if it does not involve a death. The ISM will determine the degree to which the adverse events is thought to be related to the study intervention and whether any additional action is required in response to the event. In addition to reviewing reportable adverse events, the ISM will review the prespecified safety outcomes in aggregate every six months. These safety outcomes include psychotomimetic side effects, the Clinical and Adverse Events Checklist, RASS scores during the infusion, and significant hypertension and tachycardia during the infusion.

### Ethical considerations

This study has been approved by the Human Research Protection Office at Washington University (approval #202201107) on February 15, 2022. Any protocol modifications will be approved by this IRB, and significant modifications will be communicated by updates to the trial registration and/or direct communication with participants. The study has also been registered at ClinicalTrials.gov ( NCT05233566, posted February 10, 2022). All participants will provide written, informed consent prior to their involvement in research activities. Participants will be informed that their participation is voluntary, that they may withdraw from the study at any time, and that declining to participate will not adversely affect their planned surgery or any other aspect of their medical care. The informed consent form that will be used and the completed SPIRIT checklist can be found as *Extended data.* ^66^

***Confidentiality***

All research activities will be conducted in as private a setting as possible. To protect against the risk of confidentiality breach, only the minimum necessary protected health information will be retrieved from the electronic health record. All paper documents will be stored behind two locked doors. All electronic data will be kept in an encrypted, password-protected environment accessible only to the research team. Study participant research data, to be used for statistical analysis and scientific reporting, will be stored on the Research Electronic Data Capture (REDCap) system managed by Washington University School of Medicine.

### Dissemination

The results of this trial will be disseminated via presentation at scientific meetings and publication in peer-reviewed journals, with potential audiences including anesthesiologists, psychiatrists, surgeons, and critical care physicians. In addition, key results will be published to ClinicalTrials.gov. For publications, authorship will be determined based on International Committee of Medical Journal Editors guidelines. Per National Institute of Mental Health data sharing policy, the deidentified dataset will be deposited to the National Data Archive after the study has been completed.

### Study status

At the time of initial protocol submission (April 21, 2022), no participants had yet enrolled in the study. As of the date of this revision (April 26, 2022), one participant has enrolled in the study.

## Conclusions

The trial described in this protocol will establish the feasibility of conducting a larger randomized clinical trial studying postoperative ketamine in patients with depression. This line of work will yield knowledge about whether a postoperative ketamine infusion can prevent or mitigate subsequent depression. Investigation of concurrent EEG markers may also yield insights regarding the effects of ketamine infusion on sleep architecture and potential mechanistic associations between sleep architecture and postoperative depressive symptoms. This knowledge will benefit future patients by informing treatment decisions that will enhance mental health following surgery.

## Data availability

### Underlying data

No data are associated with this article.

### Extended data

Open Science Framework: Ketamine for Postoperative Avoidance of Depressive Symptoms (K-PASS) Feasibility Study: A Randomized Clinical Trial. https://doi.org/10.17605/OSF.IO/TDGXK. ^66^

This project contains the following extended data:

- KPASS Informed Consent.pdf
- SPIRIT checklist for ‘Protocol for the Ketamine for Postoperative Avoidance of Depressive Symptoms (K-PASS) feasibility study- A randomized clinical trial’.pdf

Data are available under the terms of the Creative Commons Zero “No rights reserved” data waiver (CC0 1.0 Public domain dedication).

## Author contributions

Fritz: Conceptualization, Funding Acquisition, Methodology, Supervision, Writing – Original Draft Preparation

Pennington: Conceptualization, Funding Acquisition, Methodology, Writing – Review & Editing

Palanca: Conceptualization, Funding Acquisition, Resources, Writing – Review & Editing

Schweiger: Methodology, Writing – Review & Editing

Willie: Conceptualization, Methodology, Writing – Review & Editing

Farber: Conceptualization, Funding Acquisition, Methodology, Writing – Review & Editing
