# Supplementary material for: Protocol for the Ketamine for Postoperative Avoidance of Depressive Symptoms (K-PASS) feasibility study: A randomized clinical trial
Source: F1000Res. 2022 May 11;11:510. [Version 1] doi: 10.12688/f1000research.121529.1 (PMC10362376; doi:10.12688/f1000research.121529.1)
Supplement: Peer review history

# Peer review: Reviewer response for version 1

**DOI:** 10.5256/f1000research.133398.r188595

**Published:** 2023-07-21

**Authors:** Octavian Vasiliu

**Affiliations:** 1 Dr. Carol Davila University Emergency Central Military Hospital, Bucharest, Romania

This protocol for a study exploring the impact of ketamine on postoperative avoidance of depressive symptoms is interesting and may be useful for practitioners dealing with patients presenting with depressive disorders. Some aspects need to be addressed, in order to enhance the accuracy of the data this study will collect; please see below:

 According to the data on https://classic.clinicaltrials.gov/ct2/show/NCT05233566, this study is expected to enroll "neurosurgical patients", but there is no similar specification in the "Abstract" section of the submitted manuscript. Also, the duration of iv infusion is 3 h on the clinicaltrials.gov, and 8 h in the manuscript. I think it would be useful to insert in the "Abstract" the fact that tolerability and safety aspects will be monitored, also.

 Will there be no superior age limit as an inclusion criterion? Geriatric depression may have different evolution under treatment, although reports on the use of iv ketamine in this population exist- Bryant *et al.,* (2019) ^1^, Medeiros da Frota Ribeiro & Riva-Posse (2017) ^2^.

 The "pain" outcome is not mentioned in the online protocol published on the US NLM site.

 What does "past medical history of depression" mean? Please specify if only patients diagnosed with recurrent major depressive disorder (MDD) can participate, or if subjects with treatment-resistant MDD will also be enrolled. Any cut-off MADRS value at the baseline? Will partially remitted patients also be included, according to their MADRS scores? Will IV ketamine be an add-on to the currently administered antidepressant? Please specify if any type of antidepressant will be prohibited during the 14 days of the monitoring period. Will the patients present MDD with psychotic features be enrolled? Because this may be a confounding factor when interpreting the evolution of BPRS and CADSS-7 scores, for example.

 What kind of surgery procedures will be allowed? Only neurosurgical (as specified on the clinicaltrials.gov site), or just any type?

 There are some measurement tools with unclear purposes in the "Daya collection" section. If the authors wish to make a complete screening for other psychiatric disorders (including substance use disorders) - which would be highly recommendable - then they should also use a dedicated instrument, like MINI+, SCID5, or any other structured interview.

Is the study design appropriate for the research question?

Yes

Is the rationale for, and objectives of, the study clearly described?

Yes

Are sufficient details of the methods provided to allow replication by others?

Yes

Are the datasets clearly presented in a useable and accessible format?

Yes

Reviewer Expertise:

Psychiatry

I confirm that I have read this submission and believe that I have an appropriate level of expertise to confirm that it is of an acceptable scientific standard.

## References

[1] : Effects of Repeated Intravenous Ketamine in Treatment-Resistant Geriatric Depression: A Case Series. *J Clin Psychopharmacol* .2019;39(2) : 10.1097/JCP.0000000000001006 158-161 10.1097/JCP.0000000000001006 30742589

[2] : Use of Ketamine in Elderly Patients with Treatment-Resistant Depression. *Curr Psychiatry Rep* .2017;19(12) : 10.1007/s11920-017-0855-x 107 10.1007/s11920-017-0855-x 29138992

# Peer review: Reviewer response for version 1

**DOI:** 10.5256/f1000research.133398.r137483

**Published:** 2022-06-09

**Authors:** Boris D. Heifets

**Affiliations:** 1 Department of Anesthesiology, Perioperative and Pain Medicine, Stanford University, Stanford, CA, USA

This manuscript by Fritz *et al *describes a highly innovative clinical trial protocol to determine the feasibility of a larger study of the effects of postoperative ketamine infusion on subsequent depression rates and sleep architecture. Overall, the manuscript was exceptionally clear and well written. The feasibility outcome measures are very reasonable and well considered, and I very much appreciate this stepwise approach to clinical trial design prior to launching a much larger trial. I think this merits indexing and discussion. I do have several comments on some of the trial design components which the authors may wish to address prior to indexing, as I suspect other readers may have similar reactions.

**Patient selection**

- The authors will enroll patients with planned admission to an ICU after surgery. I assume the ICU criterion exists to accommodate the requirement for an 8 hour ketamine infusion, which in many centers can only be performed in some care areas. Have the authors done a preliminary assessment of what types of surgery they are likely to encounter? Postoperative depression in cerebral aneurysm repair and AVR patients, for example, might reflect quite different pathogenesis (eg prolonged frontal lobe retraction, long pump times & neuroinflammation).
- Are patients on high dose opioids included in this trial? Is there any issue with randomizing chronic pain patients undergoing ICU-level surgery to ketamine vs placebo when there is an arguably independent indication for intraoperative ketamine as a multimodal opioid-sparing agent?
- Authors are measuring alcohol use (AUDIT) as a predictor of outcome, will they be recruiting patients with alcohol use disorder? Withdrawal in the postoperative period, even if it does not require treatment, may significantly influence mood ratings.
- Patients with a history of depression are being recruited, but there is no mention of whether they have to meet a symptom severity criterion (eg minimal MADRS). As the authors are likely aware, the majority of patients with a chart diagnosis of depression or using an antidepressant may only have mild or moderate symptoms of depression. Therefore, the change in depression score that the authors are likely to see may be quite small and may require large sample sizes.

**Treatment paradigm**

- As described, the placebo control is unlikely to result in effective blinding given the fairly obvious effects of ketamine. I appreciate the difficulty in coming up with an active placebo that one can infuse for 8 hours without impacting patient care, so changing the comparator to ketamine may not be realistic. Given the role of expectancy bias and placebo effect, authors may wish to measure expectancy bias prior to treatment (eg Stanford Expectations of Treatment Scale). Re: measuring the blind, blinding should ideally also be assessed immediately after dosing – patients asked 24h post infusion may incorporate their response to treatment as part of their guess.
- Not sure I agree with the PK calculations – empirically, TCI methods (see https://stanpumpr.io) which incorporate a 3 compartment model (Domino *et al* 1984 Clin Pharm Ther ^1^) predict blood levels very accurately (Bowdle et 1998 Anesthesiology ^2^, Vogt *et al* 2021 Anesthesiology ^3^). Plugging into STANPUMP online the same parameters in the proposed study (3mg/kg/hr * 10 min then 0.3mg/kg/hr for 8 hrs) gives a steady state concentration of about 180ng/mL. It’s not obvious that this will be clearly inferior to a steady state of 225ng/mL, but the authors may want to take this information into consideration.

**Outcome Measures**

- Why are the authors measuring MADRS change from baseline (preop) compared to postop? Why not take the baseline measure as their last pre-ketamine score? Is it hypothesized that postoperative depression presents nearly immediately after surgery? The confound, potentially, is that patients' moods may be driven by the stress of upcoming surgery, so the authors may find that patients appear to improve before they are even randomized.

Is the study design appropriate for the research question?

Yes

Is the rationale for, and objectives of, the study clearly described?

Yes

Are sufficient details of the methods provided to allow replication by others?

Yes

Are the datasets clearly presented in a useable and accessible format?

Not applicable

Reviewer Expertise:

Perioperative mental health, translational neuroscience

I confirm that I have read this submission and believe that I have an appropriate level of expertise to confirm that it is of an acceptable scientific standard.

## References

[1] : Ketamine kinetics in unmedicated and diazepam-premedicated subjects. *Clin Pharmacol Ther* .1984;36(5) : 10.1038/clpt.1984.235 645-53 10.1038/clpt.1984.235 6488686

[2] : Psychedelic effects of ketamine in healthy volunteers: relationship to steady-state plasma concentrations. *Anesthesiology* .1998;88(1) : 10.1097/00000542-199801000-00015 82-8 10.1097/00000542-199801000-00015 9447860

[3] : Midazolam and Ketamine Produce Distinct Neural Changes in Memory, Pain, and Fear Networks during Pain. *Anesthesiology* .2021;135(1) : 10.1097/ALN.0000000000003774 69-82 10.1097/ALN.0000000000003774 PMC824934633872345
